# Subclinical *Plasmodium falciparum* Infection and HIV-1 Viral Load

**DOI:** 10.3201/eid1302.060573

**Published:** 2007-02

**Authors:** Kimberly C. Brouwer, Lisa B. Mirel, Chunfu Yang, Renu B. Lal, Margarette S. Kolczak, Anne M. Van Eijk, John Ayisi, Juliana A. Otieno, Bernard L. Nahlen, Richard Steketee, Ya Ping Shi, Altaf A. Lal

**Affiliations:** * Centers for Disease Control and Prevention, Atlanta, Georgia, USA; † Kenya Medical Research Institute, Kisumu, Kenya; ‡ New Nyanza Provincial General Hospital, Kisumu, Kenya; § World Health Organization, Geneva, Switzerland

**Keywords:** HIV, malaria, coinfection, viral load, progression, children, letter

**To the Editor:** Studies indicate that *Plasmodium falciparum* infection increases HIV replication in adults (*1*,*2*). Although malaria-related illness and death are more common in children, and HIV-1 generally progresses faster in children than in adults (*3*,*4*), to our knowledge the effect of intermittent malaria on HIV-1 viral load has not been directly explored in children. To investigate this issue, we monitored HIV-positive infants from a 1996–2001 birth cohort study in Kisumu, Kenya, a *P. falciparum*–holoendemic area.

Study design and methods have been described elsewhere (*5*,*6*). Twenty-four children that were perinatally infected with HIV were included in this substudy. During monthly visits during the child’s first 2 years of life, malaria and HIV incidence were recorded (*5*,*6*). Both children with malaria-positive blood smears and those with fever but no smear result available were treated with sulfadoxine-pyrimethamine according to national guidelines. At the time of this substudy, none of the study participants were taking antiretroviral drugs.

HIV and malaria diagnoses were determined by using standard methods (*5*–*7*). To reduce the chance of including infants infected through breast-feeding, perinatal infection was defined as >2 consecutive HIV-positive tests, with the first positive PCR results by 4 months of age (*7*). The so-called baseline viral load was the premalaria value measured 1 month before the first observation in the analysis. To be included in the analysis, follow-up visits had to have data available on the current and previous months’ viral load and malaria status and occur at roughly monthly intervals at >4 months of age.

Malaria parasites were found at 53 of 146 visits in the month before viral load measurement, although at 89% of visits in which children were malaria-positive, the children’s samples had <1,000 parasites/μL, and in only 13% of visits in which children had parasitemia did they also have fever (*8*). Median number of observations per child was 7 (range 2–18). No significant demographic or clinical differences were found between HIV-positive children in this substudy and those enrolled in the full cohort (data not shown).

Clinical and demographic variables were evaluated in univariate repeated measures analysis to determine associations with log-transformed HIV-1 viral load. Age and baseline viral load were strong predictors of current load (Table). Although not statistically significant, clearing the previous month’s malaria infection was associated with a drop in viral load (Table, p = 0.09). It was not possible to distinguish between the effects of treatment versus malaria clearance because 87% of malaria infections were treated with antimalarial drugs. However, viral load increased in those incorrectly treated for malaria presumptively (Table).

**Table:** Associations with log HIV-1 viral load in infants*

Factor	No.†	Predicted beta (SE)	p value
Baseline viral load			
Log viral load per mL	146	**0.65 (0.09)**	**<0.01**
Documented fever			
Temperature >37°C	23	−0.02 (0.11)	0.85
Previous visit documented fever			
Temperature >37°C	18	0.07 (0.14)	0.62
Vaccination within 2 weeks of visit			
Yes	15	0.14 (0.14)	0.34
Vaccination within 2 weeks of previous visit			
Yes	18	0.09 (0.12)	0.46
Pneumonia			
Present	22	0.01 (0.13)	0.93
Anemia			
Hemoglobin <8 g/dL	25	−0.25 (0.16)	0.15
Anemia during previous visit			
Hemoglobin <8 g/dL	27	0.01 (0.13)	0.95
Antimalarial drugs received/since previous visit			
Antimalarial drugs given	59	0.02 (0.10)	0.84
Malaria status current visit			
Parasitemia positive	39	−0.07 (0.10)	0.05
Malaria status previous visit			
Parasitemia positive	53	−0.14 (0.09)	0.14
Malaria status and antimalarial drug use received/since previous visit			**<0.01**
Treated parasitemia	46	−0.11 (0.09)	0.25
Did not treat parasitemia	7	−0.002 (0.17)	0.99
Treated, no parasitemia	13	**0.28 (0.10)**	**<0.01**
Did not treat, no parasitemia	80	Reference	
Malaria dynamics (previous vs. current visit)			0.36
Cleared infection‡ (+ –)	29	−0.20 (0.11)	0.09
Reinfection/persistence (+ +)	24	−0.16 (0.15)	0.28
New infection (– +)	15	−0.18 (0.14)	0.20
No malaria‡ (– –)	78	Reference	
Age, mo			
4–9	73	**–0.39 (0.13)**	**<0.01**
10–24	73	Reference	

*Statistically significant associations (p<0.05) are indicated in **boldface**. †Total sample size was 146 follow-up visits. Number of visits with the indicated characteristic are listed to the right. Sample size was 145 for pneumonia and anemia and 144 for anemia during previous visit. ‡After multiple comparisons (Tukey-Kramer) between groups in a model of the effect of malaria on log HIV viral load adjusted for age and baseline viral load, no statistically significant differences were found although clearing an infection versus no malaria had a p value of 0.10.

After adjusting for age and baseline viral load, we assessed log_10_ HIV viral load in relation to malaria clearance, persistence, absence, or new infection using a repeated measures model with autoregressive covariance structure. No differences were statistically significant, although clearing an infection versus no malaria had a 0.22 log viral load decrease (Table, p = 0.10). When 15 malaria episodes with malaria-free visits 1 month before and after the episode were compared, mean difference (signed-rank test) in viral load “before” and “after” malaria was not significant.

Our findings suggest that low-density malaria infection may not dramatically affect plasma HIV-1 levels in infants. This finding is similar to results of studies of perinatally HIV-infected children in which, although viral loads were unavailable, number of malaria episodes did not significantly affect development of AIDS-related symptoms (*9*,*10*). While clinical malaria leads to at least short-term HIV viral load increases in adults (*1*,*2*), the effect of subclinical malaria is unclear, and even less is known about coinfection in children. Children usually have higher baseline viral loads than adults; thus, the relative effect of malaria on viral load may not be as great. To reduce the impact of passively transferred maternal antibodies, analyses were done on visits after the child was 4 months old. However, lack of fully acquired antimalarial immunity may have led to different HIV/malaria interactions than seen in adults.

Viral load increased in infants that were incorrectly treated presumptively (due to fever) for malaria (Table). Most of these children were found to have other infections. Fever in malaria-endemic areas is often assumed to be malaria-related, but delay in treatment of nonmalarial infections may be harmful in HIV-infected children

Our assessment was limited in size and duration. Furthermore, in attempting to provide optimal patient care through conducting monthly surveillance and encouraging mothers to bring children in during febrile episodes, ability to assess the effect of high-density malaria was diminished because parasitemia levels never reached clinically significant levels. Finally, because malaria was diagnosed by microscopy, rather than PCR, some subclinical malaria infections may have been misclassified as malaria-negative.

Although we found no evidence of an association between subclinical, low-density malaria and infant HIV-1 viral load, the consequences of high-density or clinical malaria need to be explored. If clinical malaria in infants increases HIV-1 viral load as it does in adults (*1*,*2*), our study underscores dual benefits of malaria treatment in the context of HIV: 1) keeping malaria in check, and 2) preventing an increase in HIV viral load. Ethical issues prevent prospective studies to assess the impact of coinfection early in life, but alternatives include using animal models or stored specimens.
